# A visually secure image encryption method based on semi-tensor product compressed sensing and IWT-HD-SVD embedding

**DOI:** 10.1016/j.heliyon.2023.e22548

**Published:** 2023-11-22

**Authors:** Zhang Shuo, Hou Pijun, Cheng Yongguang, Bin Wang

**Affiliations:** Key Laboratory of Advanced Design and Intelligent Computing, Ministry of Education, School of Software Engineering, Dalian University, Dalian, China

**Keywords:** Visually secure, Zigzag transform, Compressed sensing (CS), Semi-tensor product, Image encryption

## Abstract

The conventional approach for images encryption entails transforming a regular image into an encrypted image that resembles noise. However, this noise-like encrypted image is susceptible to drawing the attention of an attacker when transmitted through a public channel. Hence, there has been a recent surge in the interest of academics towards visually secure image encryption techniques. In a broad sense, encryption methods that include visual significance should prioritize four key elements: the resemblance between the cypher picture and the carrier image, the capacity for embedding, the attainment of good recovery quality, and resilience against many forms of attacks. To address the issues pertaining to inadequate visual security, limited resistance against attacks, and subpar quality of reconstructed images observed in contemporary image encryption and compression methodologies. This paper proposes a visually secure image encryption method based on improved semi-tensor product compressed sensing, two-way cross zigzag obfuscation, and IWT-HD-SVD embedding. Firstly, the plain image is sparsely represented in the Discrete Wavelet Transform (DWT) domain, and a two-way cross zigzag mismatch strategy is proposed to disarrange the coefficient vectors. Then the plain image is encrypted as a secret image by the improved semi-tensor product compression sensing technique. After that, IWT-HD-SVD embedding technique is proposed to embed the secret image into the carrier image to generate the final meaningful cryptographic image. This dramatically improves the visual security of the cryptographic image. Simulation results show that the quality of the decrypted image is approximately 36 dB and up to 44 dB. In addition, the cryptographic image is highly robust against common noise attacks of 0.05 %.

## 1 Introduction

Digital images have evolved into an essential online carrier for storing and sending information due to the advancements in multimedia tools and the expansion of the mobile Internet [1]. However, it is crucial to consider the numerous security issues that digital photographs may encounter in an accessible network environment, such as surveillance, tampering, robbery, and loss. Information security technologies have attracted significant interest in network transmission, medical image analysis, and military security to ensure the protection of image information [2].

Based on how cipher text is conveyed, two key subcategories of network security methods can be differentiated: image encryption and information concealment. By encrypting a photo and turning it into an encrypted image that appears as noise, image encryption techniques are commonly used for secure transmission. Numerous researchers have developed multiple encryption methods based on chaos theory [3], DNA coding [3,4], quantum computing [5,6], and meta-cellular automata [6]. However, these encrypted images are highly susceptible to attacks during storage and transportation [7]. The Information-hiding technology also protects information by embedding sensitive data into a carrier, dramatically lowering the attack risk. Therefore, information-hiding algorithms for image security hold significant research value.

Steganography is a method of avoiding detection by an attacker by hiding secret information other forms of publicly transmittable messages. Steganography can hide almost any digital content, including text, images, video, or audio. After the necessary procedures, the hidden data can be extracted at the target location. Researchers have introduced numerous approaches to improve the steganographic schemes' embedding performance. Abdulla et al. devised a method to reduce the pixel changes caused by embedding, which improves the robustness and embedding capacity of steganographic images while ensuring that the distortion of steganographic images is minimized [8].

Image information can be hidden in the improvisation domain or the transform domain. Information-hiding algorithms based on the transform domain generally have better robustness. These include the Least-Significant Bit (LSB) replacement [9,10], the Most-Significant Bit (MSB) replacement [11], singular value decomposition (SVD) hiding [12], and chunked information-hiding ones [13]. The LSB and MSB replacement algorithms have simple structures and large embedding capacity and can achieve blind extraction, but they are not robust; the SVD hiding algorithm is more robust, but the extraction needs a carrier image as a reference and cannot achieve blind extraction.

Previous researchers have combined encryption and steganography to obtain ciphertext images with visual security by first encrypting the plain-text photo and embedding the encrypted photo into a carrying picture. The double protection mechanism hides the plain-text image information more effectively. Even if an attacker successfully extracts the secret picture, the plain-text photo can also be secured by relying on the encryption algorithm. In the year 2015, Bao et al. [14] introduced the theoretical framework for visible secure picture encryption, in which the simple picture is encoded only by dislocation and pixel substitution. Then, the secret picture is encrypted using the DWTCT based on the content correlation information embedded in the carrier image. This algorithm's steganography and extraction process are not fully reversible; Hence, the decrypted image's quality is contingent upon the selection of the carrier image. The majority of picture encryption solutions that prioritize visual security are based on the architecture proposed by Bao et al. Nevertheless, this particular methodology results in an encrypted image that is four times larger in size compared to the original plain-text message. This is due to the absence of compression on the plain-text image, which subsequently necessitates a greater amount of transmission bandwidth and storage capacity.

Compressive sensing has been used to boost the capacity for secret information to be hidden in the plain-text picture, without increasing the size of the cipher text image [15,16]. The hash algorithm SHA-512 linked with the plain-text picture is employed to form the different encryption parameters in this method, rendering them highly resilient against targeted plain-text assaults. However, the carrier picture's choice impacts the decrypted image's quality because of the rounding operation in its steganography and quantization processes, resulting in a rounding error that makes the embedding and extracting methods only partially reversible.

Wang et al. [17] presented an anchoring strategy based on the LSB to mitigate this problem. To create a secret image that is 1/4 the size of the original, the plain-text picture is first compressed and perceptually encrypted, then diffused and quantized. In the embedding phase, the carrier and confidential images are processed through the Integer Wavelet Transform (IWT) and turned into binary format. Each secret pixel's 8-bit information is then divided into segments of 3-bit + 3-bit + 2-bit. The lowest 3 bits of the HL component, lower 3 bits of the LH component, and lower 2 bits of the HH component from the carrier image are each replaced.

The carrier picture following the IWT inversion resembles the secret image. Because the outputs of the selected IWT transformations are all integers and avoid rounding errors, this technique's steganography and extraction processes are fully reversible. Chai et al. [18] introduced a chunk-replacement technique in which the carrier picture is split evenly into four equally sized chunks and translated into binary form pixel-by-pixel. After the compressed-aware encryption, quantization, and compression of the plain-text image have been executed, each secret pixel is equally divided into four segments of 2-bit data. The lower 2 bits of data in each of the four carrier images are altered to generate a visibly secure cipher text picture.

Furthermore, to lessen the transmission and storage requirements for the compressed-aware measurement matrix, the semi-tensor product measurement structure was introduced in a process that employs visibly reliable cryptography by Wen et al. [19]. The Semi-Tensor Product (STP) results in a smaller measurement matrix, and LSB embedding creates a visually secure ciphertext image. Xie et al. recently suggested a new half-tensor product CS model called STP-CS [20], which allows the flexible use of reduced matrices for data compression and rebuilding. This algorithm's hidden information can be extracted even without visual aids. The plane image does not influence the effectiveness of the reconstructed photo because both the embedding and extraction processes are reversible. However, the performance loss is mainly worse in noisy environments.

Zhu et al. [21] and Ye et al. [22] obtained visually secure cryptographic images using singular value decomposition to increase the robustness of cryptographic images. They apply the singular value of the secret picture to control and modify the singular quality of the carrier photo, thereby embedding secret information. The secret and carrier pictures are decomposed separately using SVD. Compared to the widely used SVD steganography algorithm, this method demonstrates higher anchoring strength However, the receiver must obtain the two matrices of the SVD decomposition of the secret image in advance when extracting, and this can consume additional bandwidth. In addition, the algorithm is implemented using the DWT, and there is no way to reverse the embedding or extraction operations. Therefore, the algorithm needs further improvement. In order to address the issues of inflexible LSB embedding and the low strength of SVD embedding, Wang et al. [22] used Bessel curve embedding to achieve meaningful image encryption. However, the use of 1DCS (1-dimensional compressive sensing) [[17], [18], [19], [20], [22], [23], [24]] to process 2D digital image signals can lead to an excessive storage computational burden [25]. Chai et al. [26] compressed and encoded simple images, embedding the secret image information within. Huo et al. [27] modified the carrier picture's wavelet coefficient matrix, significantly enhancing the compression efficiency and reconstructing the image quality.

To address the high memory space required by the measurement matrix, low attack resistance of cryptographic pictures, and insufficient reconstructed image quality, our study has sought a visibly secure picture encryption strategy based on the IWT-HD-SVD method, two-way crossed zigzag confusion, and perception of semi-tensor product compression. The compressed cryptographic picture is inserted in the cover image using the IWT-HD-SVD approach to generate a visually secure cryptographic graphic. This document presents the contributions that have been made.

(1) This research study presents a novel embedding technique named IWT-HD-SVD, which aims to enhance the robustness of the system while maintaining the imperceptibility of the encrypted image.
(2) Furthermore, this study proposes a novel two-way cross-zigzag scrambling technique as a means to enhance the level of randomness inside the elements, thereby bolstering the overall security of the encryption system.
(3) The present study incorporates singular value decomposition and column vector unitarization optimization techniques into the measurement matrix of semi-tensor product compressed sensing, with the aim of improving the quality of decrypted images. As a result, the optimized decrypted images display a 3 dB improvement in quality compared to the unoptimized procedure.

The ensuing sections of the paper are structured in the following manner. Section 2 provides an overview of the core principles behind chaotic systems and the concept of semi-tensor product compression perception. Section 3 provides an in-depth analysis of the suggested image encryption approach and its associated methodologies. Section 4 provides an account of the simulation outcomes, which is subsequently followed by a comprehensive analysis in Section 5. Finally, Section 6 serves as the concluding part of the study.

## 2 Background knowledge

### 2.1 An enhanced two-dimensional logistic chaotic model

Although the Logistic chaos has good chaotic properties, The present chaotic system exhibits limitations in terms of a limited key space and inadequate unpredictability, consequently, in order to have a more extensive key space, a higher Lyapunov exponent and better chaotic performance, and combined with the characteristics of classical Logistic chaos, a novel chaotic structure that has better properties is proposed [28]. The formula for improving the model is shown in Equation (1):

(1) $$\left\{ \begin{array}{c} X_{n+1}=\sin\left(\pi (4aX_{n}(1-X_{n}\right))+\left(1-a\right)\sin\left(\pi Y_{n}\right))\\ Y_{n+1}=\sin\left(\pi (4aY_{n}(1-Y_{n}\right))+\left(1-a\right)\sin\left(\pi X_{n+1}^{2}\right)) \end{array}\right.\text{.}$$

where $X_{n}$, $Y_{n}$ are chaotic sequence values; sin (∙) is the sine function. When a ∈ [0, 1], the chaotic framework is in a state of chaos. This chaotic system adds a sine function and new variables to the original traditional logistic chaotic system to construct an enhanced two-dimensional logistic chaotic model. and new variables to construct an enhanced two-dimensional logistic chaotic model. The system can broaden the key space, simplify the structure, and generate chaotic sequences with high pseudo-randomness.

### 2.2 Compressive sensing

In 2006, the Compressive Sensing (CS) hypothesis was proposed, demonstrating that a sparse signal might be compressed at a speed lower than the Nyquist rate while still permitting reliable recovery of the input signal [29]. Consider the length N of a signal x. Implement the sampling procedure according to Equation (2):

(2) y=Φx.

Φ is a measurement matrix, and the element y is compressed [30]. A transform domain most often causes the non-sparse native signal to become sparse. As a result, Equation (3) represents the sparsization process of signal x:

(3) x=ψs

ψ denotes a sparse orthogonal basis where s is the transformation coefficient vector in ψ.

In this process, signal compression is crucial in determining whether the signal can be recovered accurately. Therefore, the research focused on designing the observation matrix with good performance. By overcoming an optimization constraint, it is feasible to calculate the signal reconstruction [31]. The signal reconstruction process is shown in Equation (4):

(4) $$\min\|{s\|}_{1}s.t.y=\Phi \psi s\text{.}$$

The symbol $\|{s\|}_{1}$ represents the L1-norm of vector s. Several reconstruction techniques, including Orthogonal Matching Pursuit (OMP), Smoothed l0 norm (SL0), and others, have been suggested as potential solutions to address this issue. and Φ is a measurement matrix of size M × N, Φψ is a sensing matrix, ψ is a sparse basis or a sparse dictionary.

### 2.3 STP-CS

Traditional CS methods address the sampling limitation imposed by the Nyquist sampling theorem. However, when dealing with big-sized images, the measuring matrix required might become exceedingly massive. Consequently, its utilization is limited to machines possessing substantial storage capacity and computational capabilities. In their work, Peng (31) introduced the notion of Semi-Tensor Product Compressed Sensing (STP-CS). This approach combines the Semi-Tensor Product (STP), a matrix multiplication theorem designed for matrices with mismatched dimensions, with the conventional Compressed Sensing (CS) technique. The utilization of STP-CS technology results in a reduction in the dimension of the measurement matrix, leading to enhanced memory efficiency and computing performance. Set matrices $A=R^{m\times n}$ and $B=R^{p\times q}$. Let the least frequent number between n and p be t=lcm{n,p}. A, and B's semi-tensor product is thus written as Equation (5):

(5) $$A⋉B=\left(A⊗I_{t/n})(B⊗I_{t/p}\right)\text{,}$$

where, the symbol ⋉ represents the STP. and $I_{t/n}$, $I_{t/p}$ are identity arrays.

For signals $x\in R^{q}$ measurement matrix $\Phi \in R^{m\times n}$, the traditional semi-tensor compressed perceptual model is defined as

(6) y=Φ⋉x.

In Equation (6), if the number of columns in matrix Φ does not equal the number of rows in the sparse vector X, the traditional matrix multiplication operation does not work. However, the semi-tensor product has no restriction on the number of dimensions of Φ and x. If the dimensions do not match, then the operation can be performed by Equation (7) [32]:

(7) $$y=\left(\Phi_{m\times n}⊗\Phi_{q/n}\right)x\text{.}$$

The matrix $\Phi_{m\times n}$ is a measurement matrix with dimensions m × n, and the matrix $\Phi_{q/n}$ is a matrix with dimensions q × n.

## 3 Methods

### 3.1 Improved semi-tensor product compression sensing

As stated by Ref. [33], the utilization of semi-tensor product compression sensing results in a reduction in the dimensions of the measurement matrix, leading to savings in storage capacity and improved computing efficiency. However, with a compression ratio of 0.25, its image reconstruction quality is low, thus we present an improved semi-tensor product compression sensing, seeking to increase image reconstruction accuracy by increasing the column independence of the measurement matrix.

In order to achieve precise signal restoration using the reconstruction algorithm, it is imperative that the column vectors of the observation matrix demonstrate a distinct level of linear independence. Furthermore, it is worth noting that the quality of signal reconstruction at a given sampling rate is directly proportional to the strength of linear independence among the column vectors of the observation matrix [26]. Furthermore, as the minimal singular value approaches zero, the matrix's column vectors become totally linearly coupled. As a result, it is possible to optimize the observation matrix by increasing its minimal singular value without affecting the features of the original matrix.

The observation matrix undergoes a singular value decomposition, followed by the adjustment of the singular values in order to directly boost the minimal singular value of the matrix. This process therefore improves the column independence of the measurement matrix. The Singular Value Decomposition (SVD) solely takes into account the independence of the column vectors of the observation matrix, without considering the independence of the row vectors of the observation matrix. Theoretical analysis and empirical research trials provide evidence that the orthogonality of the row vectors in the observation matrix can enhance the accuracy of signal reconstruction. Consequently, we use the practice of normalizing each column of the measurement matrix as a means to improve the accuracy of reconstruction. Algorithm 1 illustrates the particular procedure.

**Table:** 

Algorithm 1 Measurement matrix optimization process.
Input: measurement matrix M
Output: Optimized measurement matrix M''.
(1) SVD optimization
[U_M_, S_M_, V_M_] = svd (M, 'econ')
vec = diag (S_M_)
M = mean (vec)
vec = M
S_M_ = diag (vec)
M' = U_M_ * S_M_ *V’_M_
(2) column vector unitization
[p, q] = size (M′);
for j = 1: n do
A (1, j) = norm (Phi (: j));
end for
A = repmat (A, m,1);
M'' = M’./A;

### 3.2 Two-way crossed zigzag confusion

**Fig. 1:**
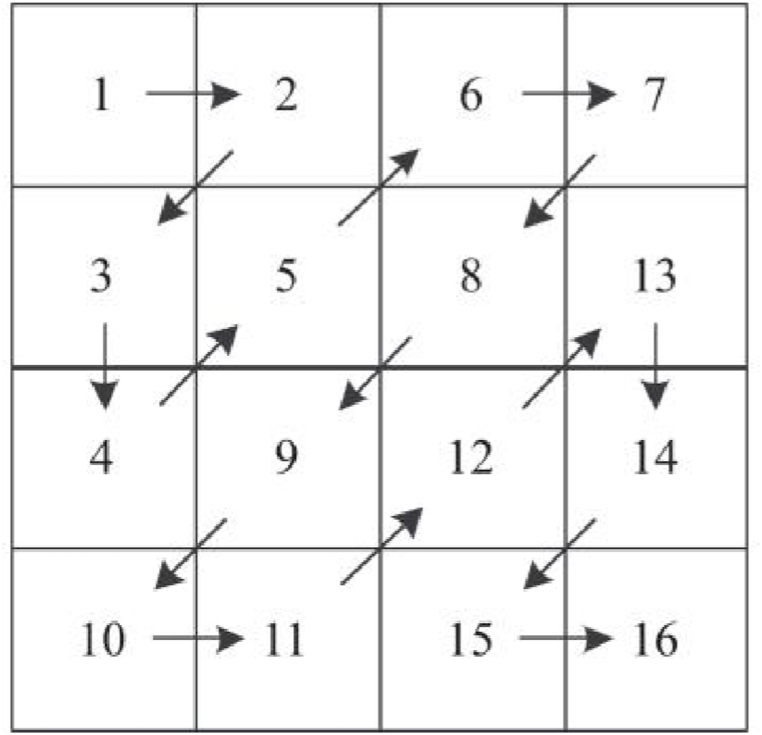
Zigzag permutation.

The zigzag permutation technique is a method utilized for scrambling data. It involves scanning the elements of a matrix in a zigzag pattern, starting at the upper left corner. This technique is frequently employed in graphic encryption due to its straightforward implementation and low time complexity. The Zigzag traversal technique is illustrated in Fig. 1 using a 4 × 4 matrix as an example. The classic positional zigzag traversal, on the other hand, has restrictions. The values of certain distinct elements inside the matrix remain unchanged while undergoing multiple instances of disorder, rendering them vulnerable to unlawful cracking by users. Furthermore, the first and last parts of the series are always the same. To address these concerns, this research suggests a two-way crossed zigzag confusion [34].

**Fig. 2:**
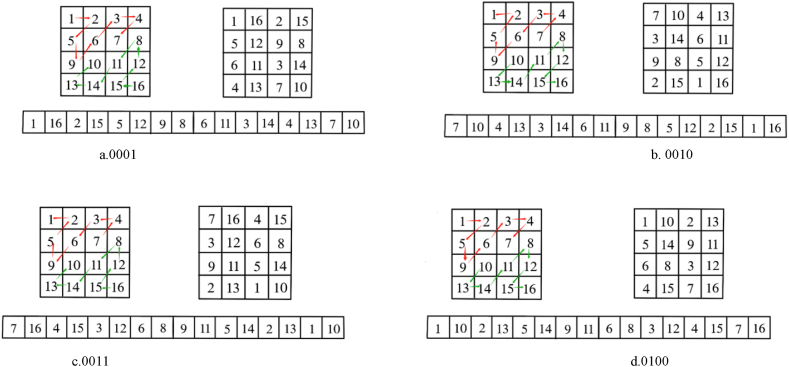
(a)–(d) Four types of dislocation with forward and reverse zigzags in the upper and lower parts.

Let the matrix dimension be 2n*2n. We divide the matrix into the diagonal upper and lower parts, perform zigzag permutations in the forward and reverse directions, and then arrange the pixels in a cross-combination to generate the permuted matrix. The matrix may be partitioned into two distinct sections, commencing from the upper-right and lower-left corners (i.e., the matrix is divided using the other diagonal so that eight types of permutations can be obtained in the end). Fig. 2 (a) -(d) show four methods of partitioning matrices using principal diagonals, respectively.

**Fig. 3:**
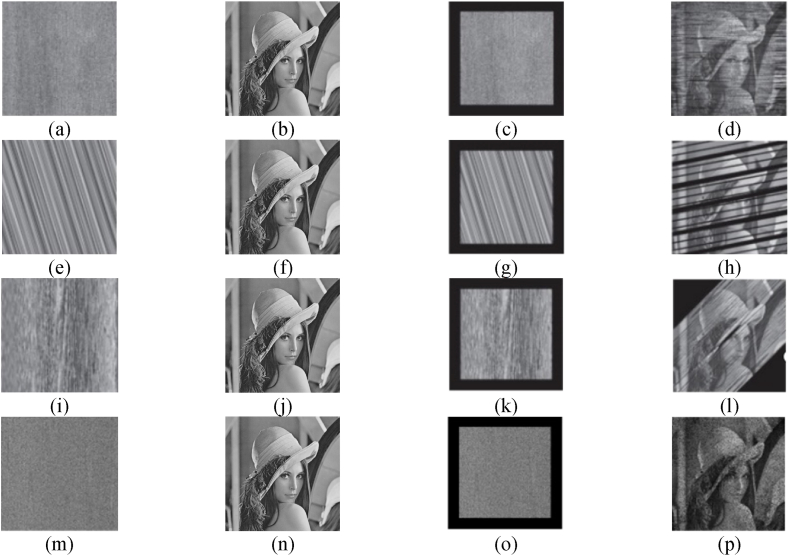
Performance comparison of several scrambling algorithms. (a) (e) (i) (m) encrypted picture, (b) (f) (j) (n) original graphic, (c) (g) (k) (o) cropped encrypted photo, and (d) (h) (l) (p) decoded picture.

Using a Lena photograph as an instance, the dislocation index proposed in Ref. [34] shows that a portion of the dislocated image is cropped off. That portion refers to the part with a width of 50. As a result, the frequency qi of the clipped portion (i.e., the portion with a pixel) that appears in the decrypted picture can be calculated. Next, the distribution of the intercepted section in the decrypted picture is evaluated to investigate the dislocation algorithm objectively. The frequency of occurrence (qi) of the clipped parts within each of the 64 blocks of the encrypted photo is determined. The public value of 10 is subsequently employed in the computation of the variance D. A smaller variance is indicative of superior disarrangement capability, and reduces the probability of the distribution falling below the absolute average of 0.3525 [34]. Fig. 3 depicts the experimental outcomes. Fig. 3 (a)–(d) depict Arnold's scrambling performance. Circular displacement scrambling performance is represented by (e) - (h), zigzag scrambling performance is represented by (i) - (l), and two-way crossed zigzag confusion performance is represented by (m) - (p). The suggested two-way crossed zigzag confusion technique distributes more uniformly in the cropped decrypted image, indicating that it beats the existing algorithms in terms of disambiguation performance. The computational findings are presented in Table 1, from which it can be seen that the suggested two-way crossed zigzag permutation has the smallest variance and the most uniform permutation performance compared to other schemes. This proves that our method is effective. However, the encryption algorithm is more complex, so it takes longer.

**Table 1:** Performance comparison of various permutation methods.

Scrambling method	Variance	Scrambling time
Arnold-based dislocation	0.6975	0.5335
Circular shift dislocation	0.7069	0.0221
Zigzag dislocation	5.5980	0.0167
Two-way crossed zigzag confusion	0.3884	0.8592

### 3.3 IWT-HD-SVD embedding method

**Fig. 4:**
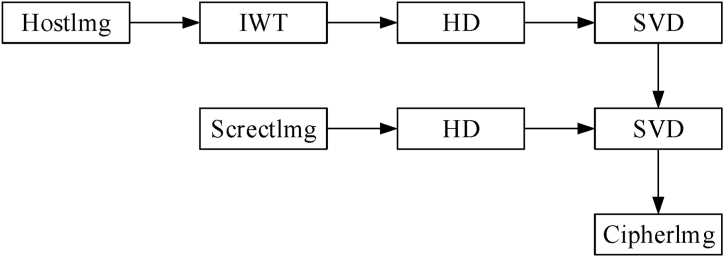
IWT-HD-SVD embedding method.

Compared with DWT, IWT increases the speed of computer operations and enables lossless image reconstruction, both features that can improve robustness and maintain imperceptibility. Singular Value Decomposition (SVD) is a powerful matrix analysis methodology, and the decomposed singular values boast better attack resistance. As a result, the transform domain and SVD-based digital watermarking algorithm is very covert and robust. If the digital image is seen as a matrix A with dimensions N × N, the singular value decomposition of A is shown in Equation (8), where $U_{A}$ and $V_{A}$ are N × N orthogonal matrices, respectively; $S_{A}$ is the N × N diagonal matrix diagonal matrix. Heisenberg Decomposition (HD) is also a standard matrix decomposition method, and the robustness is further improved when HD is used for matrix transformation. HD can be used to decompose a N × N square matrix X., as shown in Equation (9), where P denote an orthogonal matrix, and H represent an upper Heisenberg matrix. This paper combines the IWT transform with HD decomposition and SVD decomposition. To sum up. We proposed a new embedding method. Fig. 4 displays the procedure, and the specific process is shown in Algorithm 2.

(8) $$A=U_{A}S_{A}V_{A}^{T}$$

Where A is the matrix to be decomposed by SVD. The matrix $S_{A}$ is a diagonal matrix arranged in descending order, $U_{A}$ and $V_{A}^{T}$ are two unitary matrices.

(9) $$X=PHP^{T}$$

Where X is the matrix to be decomposed by HD. H is an upper Heisenberg matrix, while P is an orthogonal matrix.

**Table:** 

**Fig. 5:**
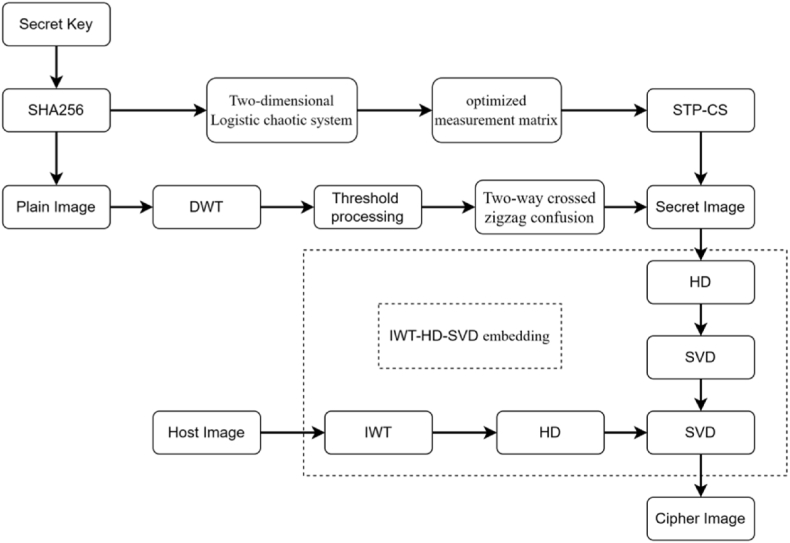
technological flowchart.

Algorithm 2 The IWT-HD-SVD embedding procedure.
Input: The secret image p'', carrier image img, embedding intensity factors α.
Output: visually secure cryptographic image C.
(1) Perform IWT on the carrier image img
[n, n] = size (img);
imgwave = liftwavedec2 (img, n,1);
a = mat2cell (imgwave, [n/2 n/2], [n/2 n/2]);
[LL,LH,HL,HH] = deal (a {:});
(2) HD-SVD decomposition of the LH part
[P, H1] = hess (LH);
[HUw, HSw, HVw] = svd (H1, 'econ');
(3) HD-SVD decomposition of secret image p''
[P1, H2] = hess (double (p''));
[Uw, Sw, Vw] = svd (H2, 'econ');
(4) Embedding at a strength of embedding factor of 0.1
Hsta = HSw + 0.1.*Sw;
(5) The visual security image C is obtained by the execution of the inverse operation.
H_hat = HUw * Hsta * HVw';
LL_hat = P*H_hat*P';
imgwave = [A, V; D, LL_hat]
C = liftwaverec2 (imgwave, m,1);

This research study presents a novel approach for secure image encryption, utilizing an enhanced semi-tensor product compression perception technique, a two-way crossing zigzag confusion mechanism, and the IWT-HD-SVD approach, all of which are combined to significantly increase security and compression efficiency. The method consists of three basic processes: improved semi-tensor product compressive sensing, confusion and IWT-HD-SVD embedding. Fig. 5 displays the complete encryption procedure.

Our method converts the sparse image into DWT sparseness before being zigzagged using a two-way cross. The utilization of Singular Value Decomposition (SVD) and the normalization of column vectors are employed to optimize the measurement matrix is obtained by the optimized logistic chaos model. The process of generating encrypted image involves the measurement, compression, and scaling of the displaced image. To create the final cryptographic picture, the encrypted photo is inserted into the carrier photo after HD and SVD have decomposed the encrypted image and IWT-HD-SVD has been employed to deconstruct the carrier image. The following diagram outlines the proposed scheme's step-by-step process.

### 3.4 Generate parameters

In the proposed strategy, hash values are used to construct the starting state of the chaotic system, which provides better defense against selective plaintext attacks and known plaintext attacks. The following are the comprehensive procedures.

Step 1 Calculate the row sum S1, column sum S2, and diagonal sum S3 of the original image matrix pixels;

Step 2 For row and S1, column and S2, diagonal and S3, and 256-bit external key K, use the hash functions MD2, MD5, SHA384, and SHA512. Next, create a new 256-bit sequence H using the SHA256 hash function. Equation (10) summarizes the above process:

(10) H=SHA256(MD2(S1),MD5(S2),SHA384(S3),SHA512(K)).

Step 3 Divide the hash value H into 32 blocks, $H=\left\{h_{1},h2,\cdots ,h_{32}\right\}$, using K and H to generate $K^{\prime }=\left\{k_{1}^{\prime },k_{2}^{\prime },\cdots ,k_{32}^{\prime }\right\}$ by anisotropy. As shown in Equation (11):

(11) $$k_{i}^{\prime }=h_{i}⊕k_{i}\text{.}$$

Step 4 Refer to Equation (12). The first 16 bits of K′ will be anisotropic to get key1, the last 16 bits will be anisotropic to key2, and key1 and key2 will be the initial values of the chaotic system:

(12) $$\left\{ \begin{array}{c} key1=k_{1}^{\prime }⊕k_{2}^{\prime }⊕k_{3}^{\prime }⊕\cdots ⊕k_{16}^{\prime }\\ key2=k_{17}^{\prime }⊕k_{18}^{\prime }⊕k_{19}^{\prime }⊕\cdots ⊕k_{32}^{\prime } \end{array}\right.\text{,}$$

### 3.5 Compression and encryption process

Step 1 A DWT transform is performed on the original graph P, after which the sparse matrix is thresholded, and the elements that fall below the threshold TS are modified to a value of 0 in order to generate the transformed matrix P'.

Step 2 The threshold matrix is divided into chunks, with each chunk size being 32*32. Two types of permutation are selected for the chunked matrix according to the remainder operation, and a two-way cross-zigzag permutation is performed; the overall matrix is permuted again to obtain p'';

Step 3 By setting Mapping a = 0.98, and iterating key1 and key2 len+1000 times, the initial 1000 sequences are subsequently discarded to remove the momentary effect of chaotic mapping, resulting in a chaotic sequence X of length len;

Step 4 Rewrite X as a 256*256 matrix. According to Equation (13), perform a tensor product operation with a unit array of dimension 2, and expand it to a measurement matrix M of 512*512:

(13) $$M=X⋉I_{2}\text{.}$$

where $I_{2}$ is a two-dimensional unit matrix, and M is the measurement matrix after dimensional expansion.

Step 5 First, apply singular value decomposition to break down the matrix using Equation (14) in order to optimize the measurement matrix, the elements in its diagonal matrix are taken out and averaged, and then the average value is assigned to the diagonal matrix. This step can improve the measurement matrix's column independence. The column vector unitization of the measurement matrix gives M″ to further improve column vector independence. Specific steps are reflected in Algorithm 1.

(14) $$M=U\Sigma V^{T}\text{.}$$

in which $\Sigma_{1}=\text{diag}\left(\partial_{1},\partial_{2},\cdots \partial r,\right)\partial_{1}\geq \partial_{2}\geq \cdots \geq \partial_{r}>0$, U and $V^{T}$ are two unitary matrices.

Step 6 By quantizing the measurement of p'' with M″, the encrypted picture p'' is acquired.

### 3.6. embedding process

3.6

Step 1 According to Equation (15), The HD-SVD decomposition of p'' yields Sw:

(15) $$\left[P,H\right]=HD\left(p^{\prime \prime }\right),\left[U_{W},S_{W},V_{W}\right]=SVD\left(H\right)\text{.}$$

where p'' is the encrypted photo. P is an orthogonal matrix and H is an upper Heisenberg matrix. $S_{W}$ is a diagonal matrix in decreasing order, $U_{W}$ and $V_{W}$ are two unitary matrices.

Step 2 To generate the LH component, the carrier picture is decomposed using IWT, and HD-SVD decomposition is performed on the LH part to obtain HSw. The above process is shown in Equation (16):

(16) $$\left[LL,LH,HL,HH\right]=IWT\left(hostImg\right);\left[P,H\right]=HD\left(LH\right);[HU_{W},HS_{W},HV_{W}]=SVD\left(H\right)\text{.}$$

where hostImg is the carrier image, and LH is the mid- and high-frequency portion of hostImg after an integer wavelet transform. ${HS}_{W}$ is a diagonal matrix, H $U_{W}$ and ${HV}_{W}$ are two unitary matrices.

Step 3 According to Equation (17), S_w_ is embedded into HS_w_ with the embedding strength:

(17) $$H{S_{W}}^{\prime }=HS_{W}+\alpha S_{W}\text{.}$$

where α is the embedding strength factor.

Step 4 Perform inverse transformation on the secret photo using Equation (18) to obtain a visually secure cryptographic image C.

(18) $$H^{\prime }=HU_{W}H{S_{W}}^{\prime }HV_{W};LH^{\prime }=PH^{\prime }P^{\prime };C=IIWT\left(LL,LH^{\prime },HL,HH\right)\text{.}$$

where C is the obtained visually secure cryptographic graphic.

### 3.7 Decryption framework

**Fig. 6:**
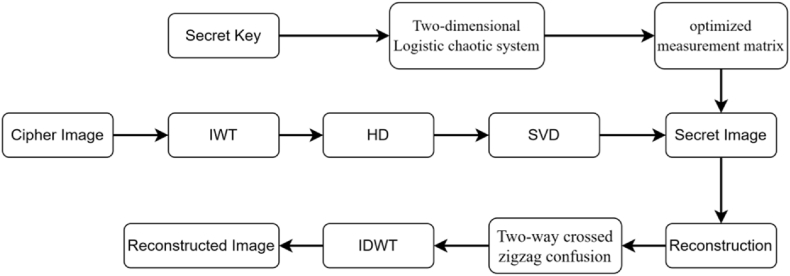
Decryption framework.

The decryption process has two fundamental phases that are the inverse of the encryption process. During the initial phase, the secret graphic is recovered from the visually secure cryptographic photo. The subsequent phase entails reconstructing the decrypted photo based on the secret picture. The secret image is first subjected to an inverse scrambling procedure, which is subsequently followed by the utilization of the SL0 algorithm to reconstruct the secret graphic. The complete decryption process is depicted in Fig. 6.

Step 1 Decompose image C at the receiver side using IWT to get four pieces and take out the LH part;

Step 2 The LH region is decomposed using HD-SVD in order to acquire the encrypted picture p''.

Step 3 The starting number of the chaotic model is derived by the processing of key, which is subsequently injected into the 2D logistic chaotic system in order to produce the measurement matrix.

Step 4 In order to acquire the final measurement matrix, do a semi-tensor product optimization quantization operation on the measurement matrix;

Step 5 Use SL0 to reconstruct the encrypted image p'' to get p';

Step 6 To obtain p, apply the inverse permutation technique to p';

Step 7 To obtain the rebuilt image, apply the inverse wavelet function on p;

## 4 Results and analysis from simulations

The proposed visually safe picture encryption technique was tested through simulations, performance tests, and evaluations. The host and popular images utilized in the testing include Lena, Clown, Fruit, Woman, Girl Face, Barbara, Baboon, Couple, Sailboat, Boat, and Airplane, which were 512*512. In addition, the parameters during the experiment were external key 7f4e9f6847484a87826d30a76b6522479b3e6128df984ad0c91b1711c52176c1, TS = 25, CR = 0.25, alpha = 0.1, and the reconstruction algorithm was SL0. All images tested in this article are from the Dataset of standard 512 × 512 grayscale test images.

### 4.1 Visual security

A laptop equipped with an i7-8550U processor was used to implement and simulate this work on the MATLAB 2020b platform to test our method's visible safety and compression capabilities.

**Fig. 7:**
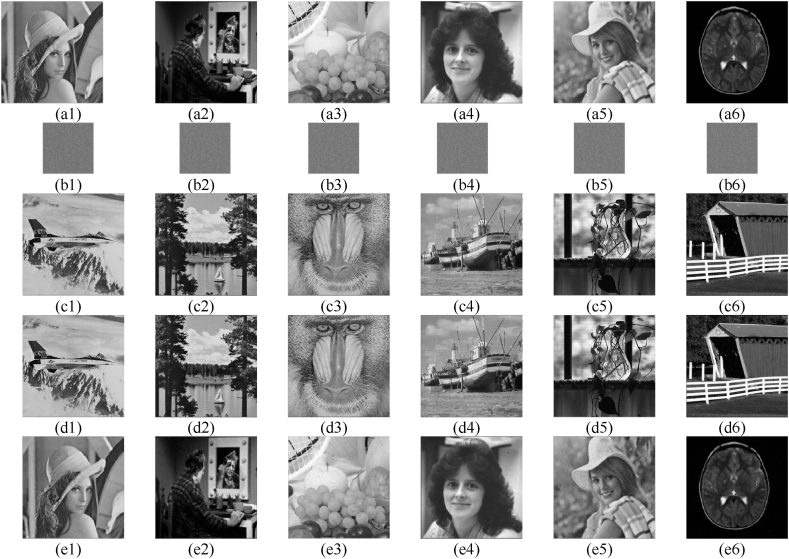
depicts the simulation results of the proposed visually meaningful encryption strategy. The original images are (a1) -(a6), the secret graphics are (b1) -(b6), the carrier photos are (c1) -(c6), the cypher pictures are (d1) -(d6), and the decrypted photos are (e1) -(e6).

The simulation findings are depicted in Fig. 7, whereby we assessed six distinct original photographs. Fig. 7 (a1) - (a6) depict the unaltered image, while (b1) - (b6) illustrate the encrypted images. The carrier image is represented by (c1) - (c6), while (d1) - (d6) symbolize the password image. Lastly, (e1) - (e6) portray the decrypted image. The secret images have a compression ratio of 0.25, resulting in a reduction in size by a factor of 1/4 compared to the plaintext images. Also, the secret images possess characteristics resembling noise. The resultant cypher image, obtained by embedding the secret graphic within the carrier photo, exhibits an identical appearance to that of the carrier photo. This effectively secures the secret picture because significant images can greatly lower the attacker's focus. The rebuilt image is of high quality and virtually identical to the original image because the embedding technique uses HD-SVD multiple decomposition to preserve the majority of the original image's information.

**Fig. 8:**
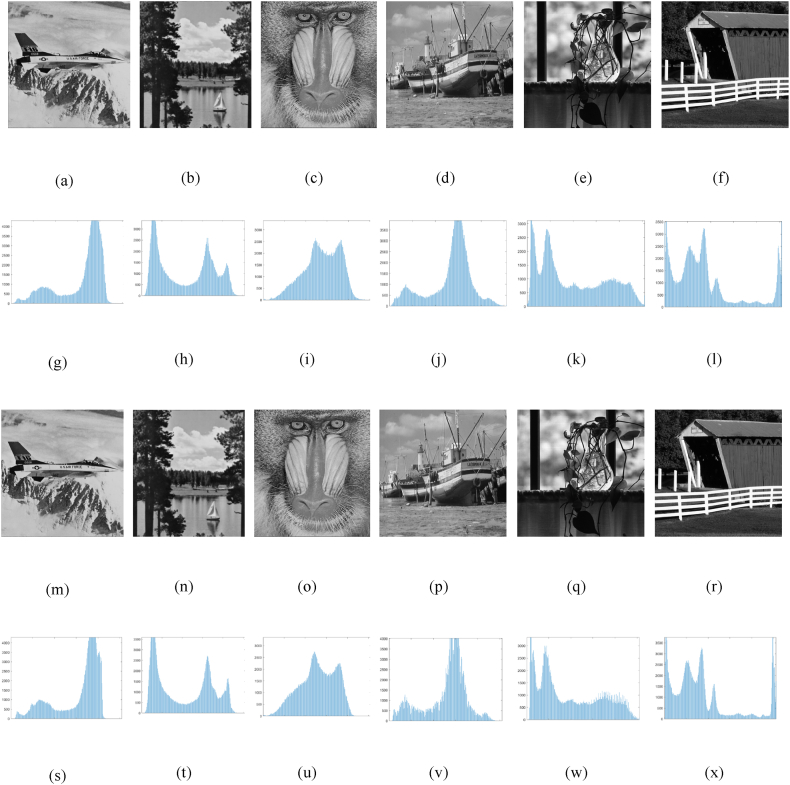
Comparative analysis of histograms between carrier images and password images. (a)–(f) carrier image, (g)–(l) carrier image histogram, (m)–(r) cryptographic image, and (s)–(x) cryptographic image histogram.

Fig. 8 illustrates the histogram test, (a) - (f) is the carrier image, (g) - (l) is the carrier graphic histogram, (m) - (r) is the cryptographic graphic, and (s) - (x) is the cryptographic photo histogram. These comparisons demonstrate that there is no noticeable visual alteration in the carrier graphic before and after the picture embedding process. This finding confirms the visual security of the cryptographic graphic.

In order to assess the efficacy of our suggested methodology, we employ the Peak Signal-To-Noise Ratio (PSNR) and Mean Structural Similarity (MSSIM) metrics, as outlined in Equations (19), (20)), respectively. These metrics are utilized to quantify the fidelity of both the encrypted picture and the reconstructed image.

(19) $$PSNR=10\times \log\frac{255^{2}}{\frac{1}{M\times N}\sum_{i=1}^{M}\sum_{j=1}^{N}{\left(D_{i,j}-I_{i,j}\right)}^{2}}$$

where M × N is used to represent the dimensions of the picture, and the pixel values at position (i, j) for the plain photo and decrypted graphic are denoted as D (i, j)and I (i, j) accordingly.

(20) $$\left\{ \begin{array}{c} SSIM\left(D_{i},I_{i}\right)=\frac{2\mu_{D}^{i}\mu_{l}^{i}+{\left(0.01\times 255\right)}^{2}}{{\left(\mu_{D}^{i}\right)}^{2}+{\left(\mu_{l}^{i}\right)}^{2}+{\left(0.01\times 255\right)}^{2}}\times \frac{2\sigma_{D}^{i}\sigma_{l}^{i}+{\left(0.03\times 255\right)}^{2}}{{\left(\sigma_{D}^{i}\right)}^{2}+{\left(\sigma_{l}^{i}\right)}^{2}+{\left(0.03\times 255\right)}^{2}}\times \frac{2\sigma_{Dl}^{i}+{\left(0.03\times 255\right)}^{2}}{2\times \sigma_{D}^{i}\times \sigma_{l}^{i}}\\ MSSIM\left(D,I\right)=\frac{1}{L}\sum_{i=1}^{L}SSIM\left(D_{i},I_{i}\right) \end{array}\right.\text{.}$$

where $\mu_{D}^{i}$ and $\mu_{l}^{i}$ represent the average values of the original photo and the decrypted picture, respectively. $\sigma_{D}^{i}$ and $\sigma_{l}^{i}$ denote the variance of X and Y, respectively, $\sigma_{Dl}^{i}$ denotes the covariance of X and Y, and C1, C2, C3 are constants.

The test results for PSNR and MSSIM are presented in Table 2. The symbol "dec" represents the numerical disparity between the initial and decrypted images. The term "cip" denotes the linkage between the visually secure cypher image and the host picture. The PSNR values for all comparisons between the cryptographic picture and the carrier image exceed 35 dB, while the MSSIM values surpass 0.9. This observation indicates a high degree of similarity between the cryptographic picture and the carrier graphic. Moreover, it is worth noting that the PSNR and MSSIM values computed between the reconstructed photos and the original images are significantly high. This observation suggests that the reconstructed images exhibit a superior level of quality. As a result, our proposed technique not only concurrently compresses and encrypts plaintext images, but also generates high-quality ciphertext images for visual security. Our proposed approach possesses the necessary attributes to fulfil the visual security, compression ratio, and reconstruction quality criteria in practical scenarios.

**Table 2:** PSNR and MSSIM values of simulation findings.

Plain image	Host image	$\text{PSN}R_{\text{cip}}$	$\text{MSSI}M_{\text{cip}}$	$\text{PSN}R_{\text{dec}}$	$\text{MSSI}M_{\text{dec}}$
Lena	Airplane	37.0971	0.9611	36.0107	0.9909
Clown	Sailboat	35.5746	0.9719	34.9702	0.9938
Fruit	Baboon	35.6100	0.9385	35.8728	0.9705
Women	Boat	35.7826	0.9544	44.3219	0.9946
Girl	Vase	36.3790	0.9763	37.3970	0.9705
Brain	House	35.9771	0.9730	35.8336	0.9790

Within this section, we proceed to assess the efficacy of the suggested methodology for encrypting the initial image through the use of diverse carrier images. The PSNR and MSSIM numbers of the cypher picture and the reconstructed photos were measured in order to assess the impact of the carrier pictures. The obtained results are presented in Table 3. It is evident that the PSNR and MSSIM values for various carrier images exhibit a high degree of similarity between the reconstructed and original graphics. Moreover, irrespective of the carrier picture employed, the cypher picture exhibits exceptional visual precision, so indicating that our proposed methodology is unaffected by the choice of carrier photo.

**Table 3:** Values of distinct carrier pictures’ PSNR and MSSIM.

Plain image	Host image	$\text{PSN}R_{\text{cip}}$	$\text{MSSI}M_{\text{cip}}$	$\text{PSN}R_{\text{dec}}$	$\text{PSN}R_{\text{dec}}$
Lena	Barbara	35.5533	0.9382	36.9917	0.9801
	Airplane	37.0971	0.9611	36.0107	0.9909
	Woman	38.4834	0.9944	34.8444	0.9906
	Fruit	37.3057	0.9716	35.4076	0.9373

### 4.2 Performance analysis

This section evaluates the suggested approach's security, robustness, and effectiveness. Then, we contrast them with currently used visually secure picture encryption techniques. It is important to note that the findings of the contrasting strategies were taken directly from the original material.

#### 4.2.1 Key security analysis

Both the range of key values and the sensitivity of the encryption method have an impact on the robustness of the encryption method against brute force attacks. The overall key space of our encryption technique is substantially more significant than the ideal key space because the length of the key parameter in the suggested scheme is 256 bits.

Next, the key sensitivity of our technique is examined using the source picture, Lena, and the host picture, Airplane. The encryption key used in the simulations is the same as that specified in Section 4, and the decryption key is decrypted by adding a small perturbation to one key at a time ($\Delta =10^{-14}$) and other important factors to obtain the upkeep.

**Fig. 9:**
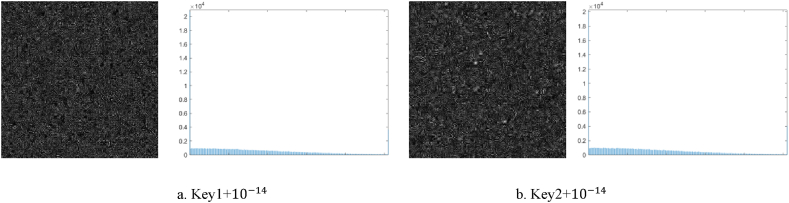
Key sensitivity analysis during decryption procedure: (a) Analysis of the graphic and its histogram decrypted by the incorrect key Key1+ $10^{-14}$; (b) Analysis of the photo and its histogram decrypted by the incorrect key Key2+ $10^{-14}$.

The visually secure encrypted images were decrypted using two sets of error keys in order to validate the experimental effect. Fig. 9 (a) - (b) respectively depict significant differences between the decryption results obtained using two different incorrect keys and those obtained using the correct key. In addition, the histogram highlights the significant visual differences between the decrypted graphic and its corresponding normal photo. In order to measure and express these variations, we utilize the Uniform Average Change Intensity (UACI) [35] and the Pixel Change Rate (NPCR) [36], as shown in Equations (21), (22)), respectively. As presented in Table 4, The statistics presented in this analysis clearly demonstrate that there is a significant difference of over 99.7 % in pixel values between the original photos and those that have been erroneously decoded. Additionally, the Unified Color Assessment Index (UCAI) closely approximates the standard value, measuring at approximately 35 %. Furthermore, the Mean Structural Similarity Index (MSSIM) scores for these images are found to be less than 0.05. This exemplifies the challenges faced by an attacker in deciphering valuable data from erroneously decoded photographs.

(21) $$NPCR=\frac{1}{M\times N}\Sigma_{i=1}^{M}\Sigma_{j=1}^{N}D\left(i,j\right)\times 100\%$$

(22) $$UACI=\frac{1}{M\times N}\frac{\sum (C_{1}\left(i,j\right)-C_{2}\left(i,j\right)}{255}\times 100\%\text{.}$$

where $D\left(i.j\right)=\left\{ \begin{array}{c} 1,ifc_{1}\left(i,j\right)\neq c_{2}\left(i,j\right)\\ 0,ifc_{1}\left(i,j\right)=c_{2}\left(i,j\right) \end{array}\right.$, The variables M and N represent the dimensions of the cypher graphic, and $c_{1}\left(i,j\right)$ and $c_{2}\left(i,j\right)$ denote the pixel values at (i, j), respectively.

**Table 4:** Key error between decrypted and normal images for NPCR and MSSIM.

Decrypted image	Decrypted key	NPCR (%)	UACI (%)	MSSIM
woman	Key1+ $10^{-14}$	99.7883	35.6778	0.0431
	Key2+ $10^{-14}$	99.7692	35.3602	0.0463

#### 4.2.2 Correlation analysis

To guarantee the strength of a cryptosystem, it is crucial for it to exhibit the capacity to withstand cryptanalysis attacks through the reduction of correlation between adjacent pixels. In order to examine the association between two neighboring pixels, we use Equation (23) to calculate the correlation coefficient CC for analysis:

(23) $$CC=\frac{E\left(X-E\left(X\right)\right)E\left(Y-E\left(Y\right)\right)}{\sqrt{D\left(X\right)}\sqrt{D\left(Y\right)}}$$

**Fig. 10:**
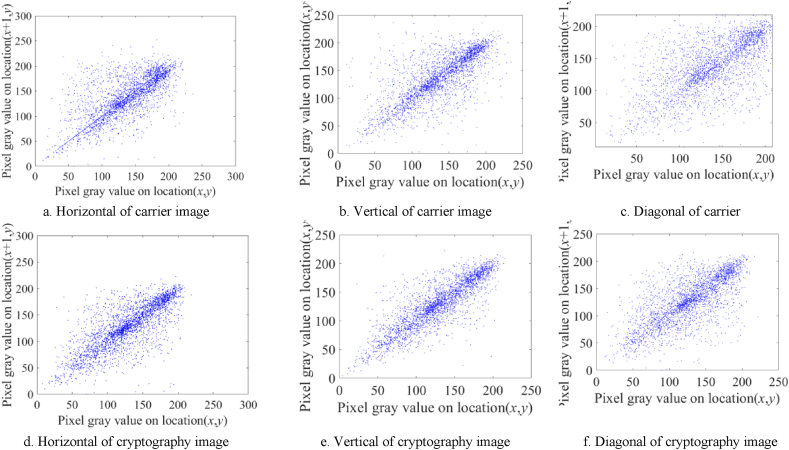
Carrier image's pixel correlation analysis is shown as a–c; the cryptography image's pixel correlation analysis is shown as d–f.

Let x and y be variables that represent adjacent pixels. E(x) and D(x) are used to signify the expected value and standard deviation of x, respectively. In this particular situation, the carrier graphic of a baboon and the original photo of Lena are utilized. Subsequently, a correlation analysis is performed on adjacent pixels by employing a random sampling technique to select 3000 pixels from the original graphic, secret photo, carrier picture, and cypher photo. Fig. 10(a)-(c) depict the correlations of the carrier picture along the horizontal, vertical, and diagonal directions, respectively. Conversely, Fig. 10(d)–(f) illustrate the correlations of the cryptographic picture. The correlation distributions of the carrier picture and the cypher photo demonstrate resemblances in the horizontal, vertical, and diagonal orientations, as illustrated in Fig. 10. The visual similarity between the cypher picture and the carrier image can be deduced from the correlation distributions stated above.

The numerical data are presented in Table 5. The frequently employed visual representation is the image commonly referred to as "Lena," while the image serving as the host or carrier is known as "Baboon." The observed correlation coefficient of the undisclosed graphic exhibits a proximity to zero. This observation implies a weak correlation between the adjacent pixels in the secret picture and the secret photo itself. Moreover, it is evident that the password image demonstrates a substantial correlation coefficient with the carrier image, suggesting a notable amount of resemblance between the two images.

**Table 5:** Correlation indices for various images.

	Plain image	Secret image	Host image	Cipher image
Horizontal	0.9658	0.0146	0.7540	0.7738
Vertical	0.9696	0.0036	0.8540	0.8480
Diagonal	0.9409	−0.0090	0.7000	0.7228

#### 4.2.3 Differential attack

The differential attack is the tactic of choice for attackers. This research investigates the association between the source photo and the encrypted picture by analyzing the encrypted image produced from two almost identical photographs that vary by a single pixel. Afterwards, the concealed information contained inside the cryptographic image is deciphered without the need for the encryption key. The quantification of our system's resilience against differential attacks is accomplished in this section by employing the Number of Pixel Change Rates (NPCR).

Hence, the Lena plain-text image is subjected to experimentation wherein a pixel value is altered to evaluate the effectiveness of the differential attack. Subsequently, the plain-text photo and the modified pixel graphic are encrypted to assess the NPCR and MSSIM of the resulting cipher-text images. Table 6 illustrates that the pixel value of the pixels located at row 255, column 270 in Lena's image, incremented by 1, is denoted as (255, 270) +1. Conversely, the pixel value of the pixels situated at row 124, column 245, decremented by 1, is represented as (124, 245) −1. The experiments involved four original plaintext images, each with a dimension of 512 x 512 pixels. These images were encrypted and then incorporated into the carrier graphic "Airplane," with the adjusted pixel values. Based on the statistics provided in the table, it is evident that when the cumulative pixel values of the two original photos are equal, the Normalized Pixel Change Rate (NPCR) values of the resulting encrypted images are approximately equal to 1. Additionally, the MSSIM value approaches 0. These findings suggest that the encryption algorithm exhibits favorable sensitivity to changes in plaintext and demonstrates effective resistance against differential attacks.

**Table 6:** Plain-text correlation.

Coordinate	Plain image	Host image	NPCR	MSSIM
(255, 270) +1 (124, 245) −1	Lena	Airplane	99.8009	0.0764
(12, 50) +1 (64, 80) −1	Dollar		99.7719	0.0887
(257, 438) +1 (356, 510) −1	Baboon		99.7971	0.0590
(249, 345) +1 (134, 10) −1	Fruit		99.8180	0.0600

#### 4.2.4 Robustness analysis

**Fig. 11:**
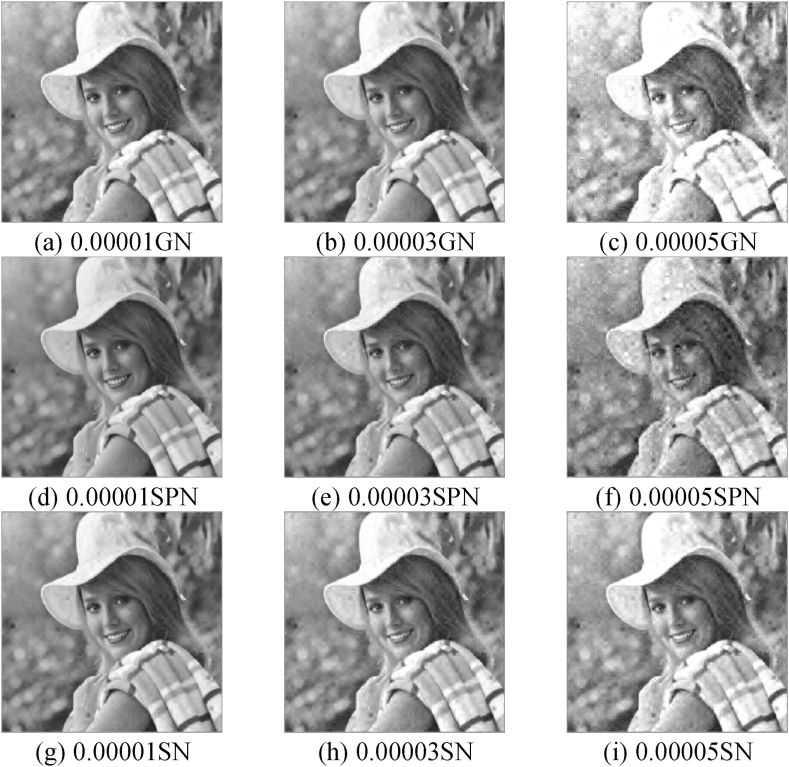
Decryption results of different attacks: (a)–(c) Decrypted images of cryptographic images subjected to 0.00001–0.00005GN noise attacks; (d)–(f) Decrypted images of cryptographic images subjected to 0.00001–0.00005SPN noise attacks; (g)–(i) Decrypted images of cryptographic images subjected to 0.00001–0.00005SN noise attacks.

Cryptographic images are susceptible to several forms of noise, which significantly complicates the process of recovering plain-text images. In this study, we assess the algorithm's capacity to withstand noise. The image containing the cipher-text is affected by varying levels of Salt&Pepper noise (SPN), Gaussian noise (GN), Speckle noise (SN). The simulation exhibits a range of noise intensities, specifically ranging from 0.00001 to 0.00005. The decryption outcomes corresponding to these noise intensities are visually presented in Fig. 11.

Fig. 11 (a)–(c) illustrate the impact of varying intensities of GN noise, ranging from 0.00001 to 0.00005, on the decrypted graphic quality of the carrier photo. Similarly, Fig. 11 (d)–(f) depict the decrypted graphic quality of the carrier photo when subjected to different intensities of SPN noise within the same range. Lastly, Fig. 11 (g)–(i) present the decrypted photo quality of the carrier graphic under the influence of varying intensities of SN noise between 0.00001 and 0.00005.

The PSNR and MSSIM values, which measure the quality of recovered picture in comparison to the original image, have been calculated and are presented in Table 7. The findings indicate that (1) decrypted images are visually identifiable within a noise intensity range of 0.00001–0.00005, with all encrypted images exhibiting MSSIM values above 0.8 and PSNR values exceeding 24 dB; (2) our encryption algorithm demonstrates the lowest resistance against GN; (3) the proposed image cryptosystem exhibits the highest resistance against SPN; (4) our image encryption scheme possesses some resistance to SN. In summary, the aforementioned image encryption technique exhibits strong resilience against many forms of noise-based assaults.

**Table 7:** PSNR and MSSIM values of decrypted images subjected to various attacks.

Decrypt image	Gaussian noise			Salt&pepper noise			Speckle noise		
	0.00001	0.00003	0.00005	0.00001	0.00003	0.00005	0.00001	0.00003	0.00005
PSNR	26.3021	26.2271	24.4122	25.0303	28.5783	26.6483	28.3488	25.6844	25.0056
MSSIM	0.9644	0.9682	0.8334	0.8814	0.9666	0.8892	0.9761	0.9567	0.9269

**Fig. 12:**
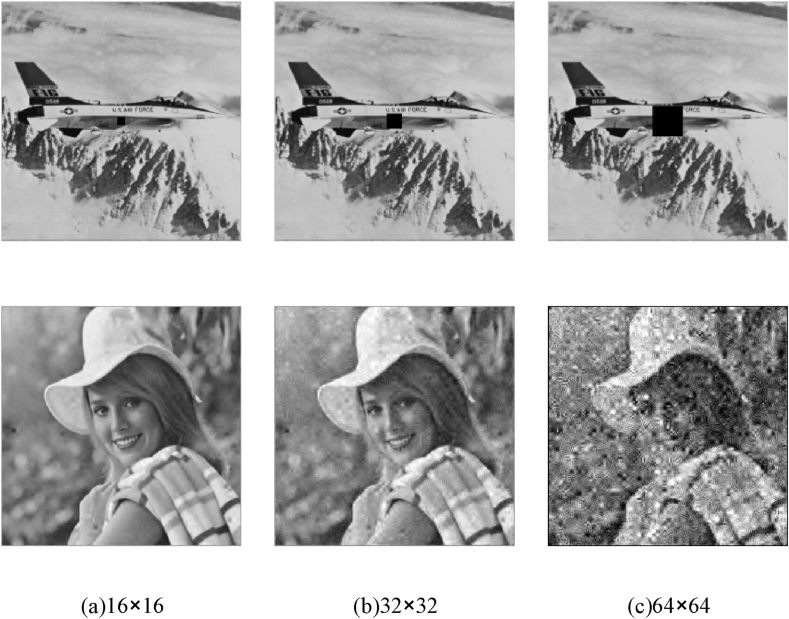
Decryption results in the face of shearing attacks:(a) Decrypted graphic with data loss of 16 × 16; (b) Decrypted graphic with data loss of 32 × 32; (c) Decrypted graphic with data loss of 64 × 64.

Furthermore, we conducted an assessment of the picture encryption system's resilience against shear attacks. The data of varying dimensions located at the center of the encrypted photo is removed, followed by the decryption of the associated encrypted image. The resulting recovered image is depicted in Fig. 12. Fig. 12 (a)–(c) illustrate the visual quality of encrypted photos when subjected to various cropping sizes, specifically 16 × 16, 32 × 32, and 64 × 64, respectively.

The illustration illustrates the encrypted picture in the first row, which has undergone varying degrees of data loss, and the decrypted image in the second row. The results of the calculations for the decrypted photo and the original photo are presented in Table 8. Based on the observations made in Fig. 12 and Tables 8 and it is evident that as the volume of lost data escalates, the decrypted photo experiences a degradation in sharpness. However, it is noteworthy that even when the cutoff size exceeds 64 × 64, the image still retains recognizable information. Upon comparing the findings with the existing literature [37], it is evident that the suggested approach yields somewhat superior image quality at an equivalent cut size. This observation suggests that the proposed technique exhibits enhanced resilience against cropping attacks.

**Table 8:** Results in the face of shearing attacks.

Size of data loss	PSNR (Ours)	Ref [37]
16*16	34.8807	30.06
32*32	29.6078	28.65
64*64	28.5027	–

#### 4.2.5 The impact of various parameters on the findings

**Fig. 13:**
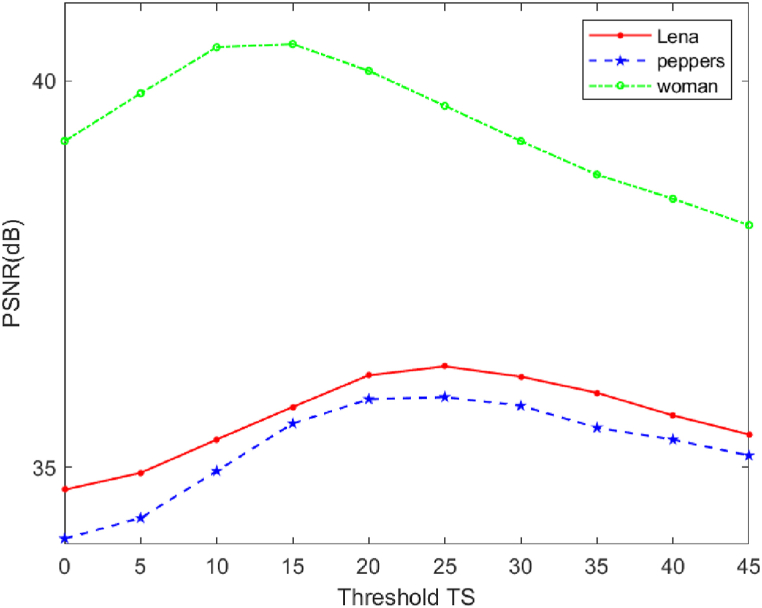
Effect of thresholding on the visible quality of decrypted images under various circumstances.

The application of thresholding techniques in picture encryption based on compressed sensing (CS) is crucial for enhancing compression efficiency. Fig. 13 depicts the influence of the threshold TS on the Peak Signal-to-Noise Ratio (PSNR) value recorded while comparing the original and decrypted images under various situations. It is clear from the figure that when TS rises, the value of PSNRdec first rises and then falls. Moreover, the image reconstruction quality is highest at around TS = 25. Therefore, the value of TS chosen in this work is 25.

**Fig. 14:**
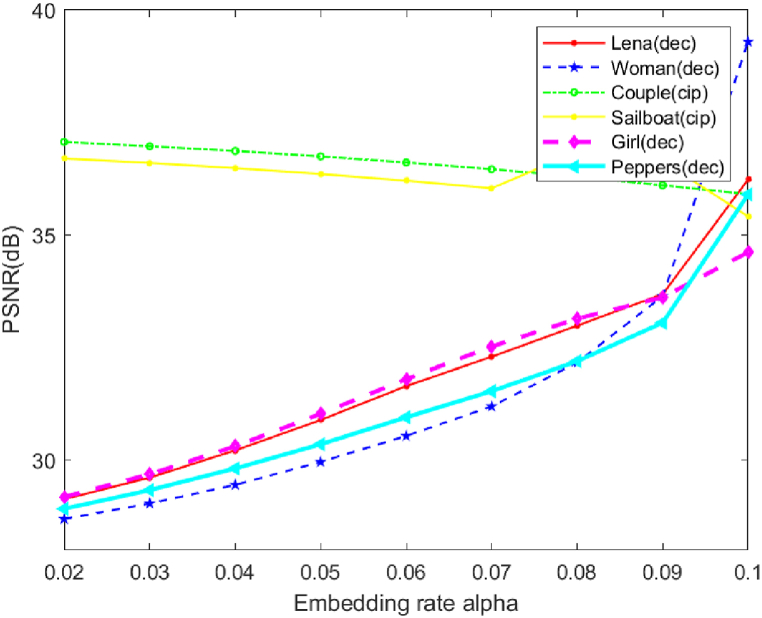
PSNR vs α with different images.

At the same time, we analyze the effect of the variation of α on the simulation results. For the purpose of experimentation, two distinct sets of photos, namely Woman and Lena, as well as Girl face and peppers, both with dimensions of 512*512, were first chosen. These images were subsequently subjected to compression and encryption processes. Subsequently, the two sets of photographs are incorporated within the carrier images, namely the couple and the sailboat. The experimental findings are depicted in Fig. 14. Based on the depicted graph, it is evident that an increase in the size of the results in a decrease in the PSNR between the carrier picture and the steganographic photo. This implies that the steganographic image exhibits reduced imperceptibility. The PSNR between the original picture and the decrypted photo exhibits a consistent pattern of incremental improvement, with a higher PSNR value indicating a more rapid rate of growth. Since the PSNR of the steganographic image produces relatively less degradation with the change of α, here we prioritize the decrypted image quality, so we set the value of α to 0.1.

The formulation of the measurement matrix constitutes a fundamental component within the field of computer science theory, exerting a direct influence on the efficacy of the reconstruction process. To achieve high-quality reconstruction, Minimizing the correlation between the measurement matrix and the sparse basis is of utmost importance. On the contrary, it is imperative to underscore and enhance the independence of the measurement matrix in order to optimize the observation matrix. The encryption algorithm employs singular value decomposition (SVD) and column vector unitization to enhance the efficiency of the measurement matrix. Initially, the SVD decomposes the measurement matrix, and subsequently, the elements of the resulting diagonal matrix are averaged. Afterwards, the elements in the diagonal matrix are assigned the average value. Ultimately, the resulting measurement matrix undergoes column vector unitization. This procedure effectively improves the degree of column independence exhibited by the measurement matrix.

**Fig. 15:**
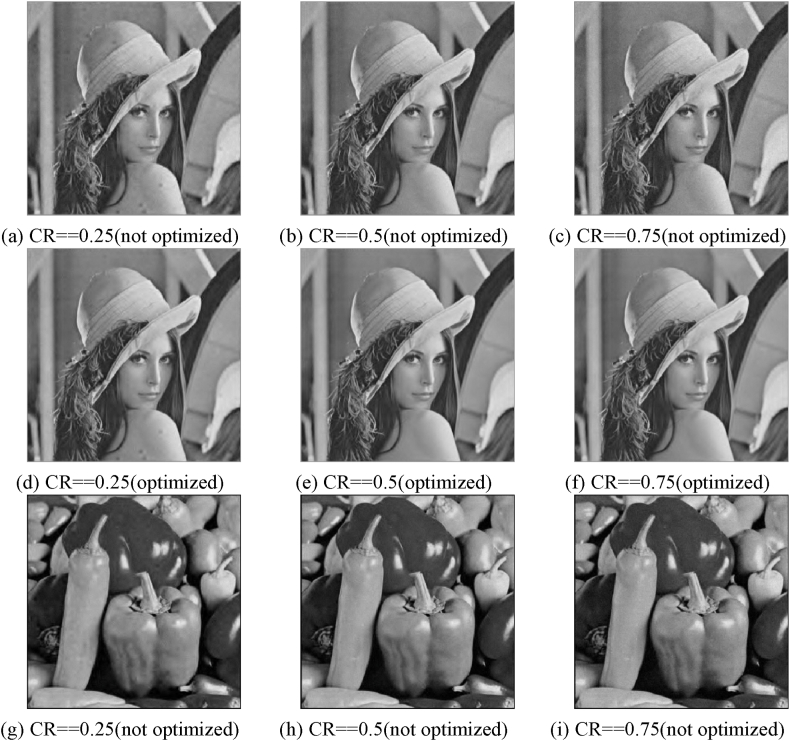

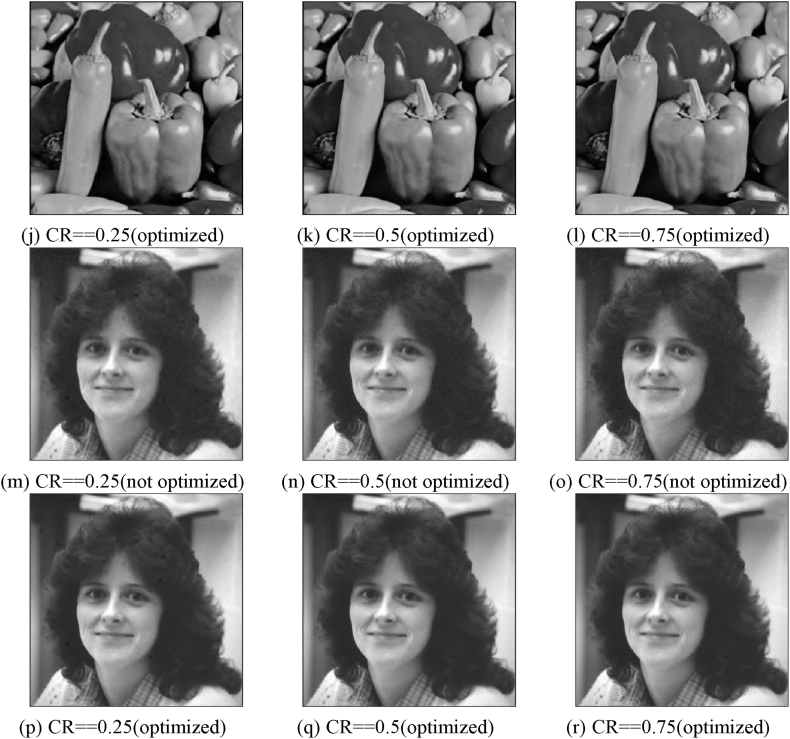
Reconstructed photos of Lena, peppers, and a woman at various CR levels: (a)–(c) Unoptimized Lena reconstructed pictures at various CR; (d)–(f) Optimized Lena reconstructed images at various CR; (g)–(i) Optimized Lena reconstructed graphics at various CR; Peppers reconstructed photos at various CR; (j)–(l) Peppers reconstructed pictures at various CR; (m)–(o) Unoptimized Woman reconstructed pictures at various CR; Optimized Woman rebuilt photos at various CR;

The experimental evaluation of the proposed optimization approach on grayscale images involves using three test images, namely Lena, Pepper, and Woman. These images have a size of 512 × 512 pixels. The reconstruction method employed in this experiment is SL0. The purpose of this experiment is to validate the efficiency of the suggested optimization method on grayscale images. Fig. 15 depicts the reconstructed pictures of test shots with varying compression ratios (CR). Fig. 15 (a)–(f) show a comparison of reconstructed images obtained by Lena before and after optimizing the measurement matrix under different compression ratios; Fig. 15 (g)–(l) show a comparison of reconstructed images obtained by Image Peppers before and after optimizing the measurement matrix under different compression ratios; and Fig. 15 (m)–(r) show a comparison of reconstructed images obtained by Woman before and after optimizing the measurement matrix under different compression.

Our research shows that improving the measuring matrix has a positive impact. To assess this optimization, we employed PSNR values as a metric, specifically focusing on the photographs of Lena, Pepper, and Woman. The findings are displayed in Table 9, Table 10, Table 11. The data shown in the table indicates a positive link between the compression ratio and the quality of picture reconstruction. Moreover, the optimization of the measurement matrix leads to a substantial improvement in image quality compared to the scenario where the matrix is not optimized.

**Table 9:** Comparison of Lena compression performance before and after measurement matrix optimization.

Lena	CR = 0.25	CR = 0.5	CR = 0.75
PSNR (Unoptimized)	32.9528	33.9770	31.0541
PSNR (Optimized)	35.8720	37.4819	37.8532

**Table 10:** Comparison of Peppers compression performance before and after measurement matrix optimization.

Peppers	CR = 0.25	CR = 0.5	CR = 0.75
PSNR (Unoptimized)	33.4215	33.4419	31.6217
PSNR (Optimized)	35.4008	36.5186	36.2695

**Table 11:** Comparison of Woman compression performance before and after measurement matrix optimization.

Woman	CR = 0.25	CR = 0.5	CR = 0.75
PSNR (Unoptimized)	35.3874	33.7902	31.6772
PSNR (Optimized)	39.1363	39.6139	39.2898

According to the data presented in Table 9, Table 10, Table 11, there is a noticeable increase in PSNR values when CR is raised from 0.25 to 0.75. Specifically, the PSNR values obtained using the optimized measurement matrix consistently exceed those obtained using the unoptimized measurement matrix. The decrypted image corresponding to the same compression ratio (CR) exhibits a higher degree of similarity to the original graphic. Specifically, the improvement in image quality for Lena's image ranges from 3 dB to 6 dB, while Peppers' image quality experiences an enhancement of approximately 3 dB–5 dB. Similarly, the image quality of Woman's image is enhanced by approximately 4 dB–8 dB.Therefore, the optimization strategy employed for the measurement matrix demonstrates efficacy.

#### 4.2.6 Contrast analysis

Within the domain of picture encryption methods that emphasize visual security, the level of similarity between the carrier picture and the cypher text photo is seen as a critical parameter. The PSNR and MSSIM values for the cypher photo and carrier picture are calculated using various methods. These values, along with the results from previous studies [37,36], are presented in Table 12. The comparison reveals that the suggested model exhibits superior cypher photo agnosticism compared to the scheme proposed in Refs. [37,36]. This conclusion is supported by numerical analysis. The cryptographic images exhibit average PSNR and MSSIM values of approximately 35 dB and 0.95, respectively. These values surpass those obtained from the systems referenced as [37,36], suggesting that our suggested method is both visually secure and effective.

**Table 12:** Comparison of visual safety.

Plain image	Carrier image	PSNR			MSSIM		
		Our	Ref. [37]	Ref. [36]	Our	Ref. [37]	Ref. [36]
Lena	Peppers	37.1733	33.3252	31.7986	0.9774	0.9032	0.9903
Airplane	Baboon	33.6406	33.0562	32.5976	0.8300	0.9586	0.9955
Girl face	Goldhill	36.0992	33.1094	32.0047	0.9729	0.9255	0.9942
Barbara	Bridge	34.2738	33.1244	31.7391	0.9388	0.9647	0.9946

Table 13 presents a comparison of several techniques in terms of reconstruction quality, as measured by PSNR values, at varying compression ratios. The picture size used for this analysis is 512*512 pixels. When the CR is set to 0.25, the suggested approach exhibits a notably higher PSNR value compared to the PSNR values reported in Refs. [[35], [38], [39], [40]]. When CR is set to 0.5, the PSNR value of our suggested algorithm surpasses that of the technique mentioned in Refs. [[35], [38], [39], [40]]. This suggests that our proposed algorithm is more suited for reconstructing larger-sized images under compression and exhibits favorable reconstruction performance. Table 14 presents a comparison between the proposed technique and the one described in the literature [21] in terms of their resistance to noise attacks. The table clearly demonstrates that the suggested algorithm exhibits consistent and exceptional performance in picture reconstruction under common noise attack settings ranging from 0.0001 % to 0.0005 %. Therefore, the technique we have proposed exhibits strong robustness and can be effectively employed for ensuring secure communication across a wide range of applications.

**Table 13:** Compression performance contrast.

CR	Plain image	PSNR				
		Ref. [40]	Ref. [39]	Ref. [38]	Ref. [35]	Ours
0.25	Lena	–	–	24.39	32.290	35.8720
0.5	Lena	0.0233	23.3608	30.71	36.268	37.4819
	Pepper	29.8787	27.2366	–	36.5198	36.5186

**Table 14:** Comparison of anti-noise performance.

Noise type	PSNR					
	0.0001 %		0.0003 %		0.0005 %	
	Ours	Ref. [21]	Ours	Ref. [21]	Ours	Ref. [21]
SPN	34.6033	30.2759	34.6033	28.5631	33.7143	27.1642
GN	34.2724	29.6062	34.2661	27.5793	34.2606	27.4337
SN	34.6033	30.2759	34.3432	30.0845	33.7753	29.0767

#### 4.2.7 Execution efficiency analysis

The suggested technique comprises two primary components, namely the encryption phase and the embedding phase, inside its algorithmic framework. During the initial stage, which encompasses the generation of the measurement matrix, the discretization of the coefficient matrix, and the linear measurement, the time complexity is denoted as $\Theta \left(C_{1}\cdot N^{2}\right)$. The time complexity incurred during the embedding component is $\Theta \left(C_{2}\cdot N^{2}\cdot logN\right)$, where the symbols *C*_*i*_ *(i* = *1*, *2)* are fixed constants. Furthermore, it is important to note that the temporal complexity of the decryption phase is heavily influenced by the specific reconstruction algorithm utilized.

The results of the comparison of encryption efficiency of various encryption schemes are presented in Table 15. The plaintext image picked for this study is "Lena," which has dimensions of 256 × 256 and 512 × 512 pixels. The table illustrates a clear positive correlation between the dimension of the plain-text picture and the corresponding operation time consumed, indicating a quick increase in the latter as the former grows. Similar to other forms of comparative literature, the process of embedding extraction is characterized by its efficiency, requiring minimal time. Conversely, the phase of rebuilding is often recognized as the most time-consuming component, mostly attributed to the intricacy of the reconstruction method. Nevertheless, the efficiency of our encryption system consistently surpasses that of the cited literature [18,37,41,42].

**Table 15:** The encryption efficiency comparison with other algorithms (Unit: s).

Item	Lena (512*512)					Lena (256*256)				
	Proposed	Ref. [37]	Ref. [18]	Ref. [41]	Ref. [42]	Proposed	Ref. [37]	Ref. [18]	Ref. [41]	Ref. [42]
Encryption	0.6287	0.8125	0.9265	0.7451	0.093751	0.2203	0.2026	0.1574	0.1462	0.025098
Embedding	0.2124	0.0234	0.9099	69.0720	1.004689	0.1827	0.0060	0.2322	16.9714	0.084601
**Total**	0.8411	0.8359	1.8364	23.2724	1.123162	0.4030	0.2086	0.3896	5.7059	0.109699
Extracting	0.1407	0.2984	1.70	34.5340	0.548270	0.0159	–	0.28	8.5617	0.044100
Reconstruction	3.9150	14.6444	6.68	27.5058	5.285881	0.9195	–	1.25	6.6999	0.964210
**Total**	4.0557	14.9338	8.38	20.6829	5.863756	0.9354	–	1.53	5.0888	1.00831

## 5 Discussion

The comparison results demonstrate that the proposed system exhibits superior visual safety and robustness to attacks when compared to the designs discussed in Refs. [37,36,21]. The main reason is that the stability of the IWT transform and HD-SVD decomposition helps to improve its visual security and attack resistance. Furthermore, in alternative literature sources, the determination of the initial number of the chaotic structure. is dependent on an external key input, which remains constant regardless of the picture and is susceptible to attacks from the chosen plaintext. In order to address this limitation, the algorithm proposed in this research study presents a novel approach where the encryption key is intricately linked to the plaintext data, and the beginning number of the chaotic sequence is not only influenced by the external key but also by the individual pixels of the plaintext picture. The alteration of pixel values in the plaintext image induces a corresponding modification in the chaotic sequence, hence enhancing its resilience against selective plaintext assaults. To address the challenge provided by high computational complexity, the proposed approach employs a low dimensional chaotic system for encryption. This strategy effectively mitigates the complexity while enhancing the encryption capabilities.

## 6 Conclusion

This study presents a novel picture encryption algorithm that ensures visual security. The proposed system is built upon an enhanced semi-tensor product compressed sensing framework and utilizes IWT-HD-SVD embedding technique. In our proposed methodology, the initial step involves the application of a two-way cross-zigzag scrambling technique to disarrange the plain photo. The encryption procedure of the plaintext images involves the utilization of the STPCS approach after undergoing scrambling. The secret graphic is embedded into the carrier photo using the IWT-HD-SVD technique, resulting in the creation of a visually secure cryptographic picture. The results derived from the simulation indicate that the structure proposed exhibits positive attributes in relation to security, decryption quality, and operational efficiency.

The solution we propose addresses the limitations seen in current visual image encryption algorithms, such as inadequate visual security, vulnerability to attacks, and suboptimal quality of reconstructed images. Furthermore, the utilization of semi-tensor product compressed sensing technique results in a reduction in the dimensions of the measurement matrix, hence enhancing the efficiency of transmission. Additionally, this approach does not necessitate the allocation of supplementary storage capacity or transmission bandwidth. The utilization of the IWT-HD-SVD embedding approach in conducting two matrix decompositions of the secret graphic alongside the carrier picture prior to embedding significantly enhances the resilience of the cypher image.

It is noteworthy to emphasize that our analysis has solely focused on the visual encryption of individual grayscale photos. Encryption of visually meaningless photos, a set number of images, and many images of varying sizes is more in line with big data requirements. However, compression-aware image dimension constraints make these criteria impossible to realize. And, while the STP-based compression algorithm effectively reduces reconstruction time, the SL0 algorithm used in this paper still requires more running time, and the image reconstruction quality of other reconstruction algorithms is not ideal, so in our future work, we hope to overcome this difficulty while working on the design and improvement of reconstruction algorithms, as well as finding a good way to embed multiple photos of different sizes into a single graphic. Better integration of visually secure image encryption algorithms with real-life information security needs. Areas we are currently exploring include transportation and medical information security, visually secure encryption of images containing sensitive user data for secure transmission and storage, and meeting people's needs for information protection in the data era.

## Data availability

Data will be made available on request.

## CRediT authorship contribution statement

**Zhang Shuo:** Writing – original draft, Data curation. **Hou Pijun:** Methodology, Formal analysis. **Cheng Yongguang:** Software, Data curation. **Bin Wang:** Writing – review & editing, Funding acquisition.

## Declaration of competing interest

The authors declare that they have no known competing financial interests or personal relationships that could have appeared to influence the work reported in this paper.
